# Improving personal continuity in general practice: a focus group study

**DOI:** 10.3399/BJGP.2024.0099

**Published:** 2024-11-26

**Authors:** Lex JJ Groot, Esther Janssen, Marjan J Westerman, Henk J Schers, Jako S Burgers, Martin Smalbrugge, Annemarie A Uijen, Henriëtte E van der Horst, Otto R Maarsingh

**Affiliations:** Department of General Practice, Amsterdam UMC, Vrije Universiteit Amsterdam, Amsterdam and Aging and Later Life, Amsterdam Public Health, Amsterdam, the Netherlands.; Department of Neurology, Radboud University Nijmegen Medical Centre, Nijmegen, the Netherlands.; Department of Epidemiology and Data Science, Amsterdam Public Health Research Institute, Amsterdam UMC, Vrije Universiteit Amsterdam, Amsterdam, the Netherlands.; Department of Primary and Community Care, Radboud University Nijmegen Medical Centre, Nijmegen, the Netherlands.; Department of General Practice, Care and Public Health Research Institute (CAPHRI), Maastricht, the Netherlands.; Department of Medicine for Older People, Amsterdam UMC, Vrije Universiteit Amsterdam, Amsterdam and Aging and Later Life, Amsterdam Public Health Research Institute, Amsterdam, the Netherlands.; Department of Primary and Community Care, Radboud University Nijmegen Medical Centre, Nijmegen, the Netherlands.; Department of General Practice, Amsterdam UMC, Vrije Universiteit Amsterdam, Amsterdam and Aging and Later Life, Amsterdam Public Health, Amsterdam, the Netherlands.; Department of General Practice, Amsterdam UMC, Vrije Universiteit Amsterdam, Amsterdam and Aging and Later Life, Amsterdam Public Health, Amsterdam, the Netherlands.

**Keywords:** continuity of care, focus groups, general practice, physician–patient relations

## Abstract

**Background:**

Personal continuity is an important dimension of continuity of care in general practice and is associated with many benefits including a higher quality of GP care and lower mortality rate. Over time, changes in society and health care have challenged the provision of personal continuity. Older patients in particular experience more negative consequences from receiving discontinuous care.

**Aim:**

To explore the perspectives of GPs, older patients, practice nurses, and assistants on improving personal continuity in general practice, and to identify barriers and facilitators that affect this improvement process.

**Design and setting:**

A qualitative study using focus groups was conducted from May to August 2019.

**Method:**

We organised four focus groups: two with GPs (*n* = 17), one with patients (*n* = 7), and one with practice assistants (*n* = 4) and practice nurses (*n* = 2). Focus groups were analysed using reflexive thematic analysis.

**Results:**

Personal continuity was viewed as being provided by the entire general practice team and not just by the patient’s own GP. It was suggested that investing in team communication and stability (for example, by efficient use of the electronic health records) and retaining the availability and accessibility of the patient’s own GP for patient care, especially for frail older persons, (for example, by delegating tasks) could improve personal continuity. Barriers and facilitators were perceived at the individual (for example, GPs’ involvement in tasks), organisation (for example, staff shortages), and societal level (for example, payment system).

**Conclusion:**

As general practice moves towards a more team-based approach to ensure personal continuity, efforts to improve personal continuity should focus on supporting team-based provision of continuous care.

## Introduction

Continuity of care is a core value of general practice.^1^^,^^2^ It includes three dimensions: personal, team, and cross-boundary continuity (Box 1).^3^

**Box 1.** Dimensions of continuity of care defined by Uijen *et al* (2012)^3^

‘Personal continuity: having a personal provider in every separate care setting who knows and follows the patient.’ ‘Team continuity: exchange of relevant patient information and cooperation between care providers within one care setting to ensure that care is connected.’ ‘Cross-boundary continuity: exchange of relevant patient information and cooperation between care providers from different settings to ensure that care is connected.’

From former studies, personal continuity is known to be highly valued by both GPs and patients and there is a growing body of evidence associating personal continuity with many benefits.^1^^,^^2^^,^^4^^–^8 These include higher patient and doctor satisfaction,^9^^–^12 higher quality of GP care,^13^^–^16 lower healthcare costs,^17^^–^19 and lower mortality rates.^18^^–^25

Over time, changes in society and health care have challenged the provision of personal continuity of care.^26^^–^28 There is an increase of older patients with chronic diseases who often receive care from multiple healthcare providers from different organisations.^29^ These patients benefit more from having personal continuity than younger, more healthy people and are more at risk for negative consequences by fragmented and discontinuous care.^8^^,^^30^^–^34 In addition, GPs may experience more difficulties in building and maintaining a personal relationship with their patients because of working part time, larger group practices, and more locum workers.^35^^–^37

We previously performed a cross-sectional survey study among 249 GPs and 582 older patients to investigate their views on personal continuity and how personal continuity can be improved.^38^ It was found that both GPs and patients still place high value on personal continuity and a wide range of suggestions to improve personal continuity in general practice was given. However, survey responses are brief, non-interactive and lack contextual information.^39^ In addition, it is important to consider the views of other members of the primary healthcare team, such as practice assistants and practice nurses. In Dutch general practice, practice assistants are the first contact for patients and responsible for planning consultations, telephonic triage, and performing supportive medical tasks. Practice nurses support the GP in the care of patients with chronic diseases, such as diabetes mellitus, chronic obstructive pulmonary disease, cardiovascular diseases, or mental health issues.^40^ To the best of our knowledge, no previous studies detailing the views of practice assistants or practice nurses on how to improve personal continuity have been conducted.

As part of the optimising personal continuity for older patients (TOOL) study,^41^ we intended to add to the wide range of suggestions of our survey study by adding a more in-depth perspective through qualitative research. The aim of this focus group study was to identify possibilities to improve personal continuity for older patients by exploring the perspectives of GPs, patients, practice nurses, and practice assistants on improving personal continuity and to identify barriers and facilitators affecting this process.

**Table:** How this fits in

Owing to changes in healthcare and society, the provision of personal continuity has moved from being provided exclusively by the patient’s own GP towards a teamoriented approach. As a result, team communication and staff stability are becoming more important for maintaining personal continuity. Retaining the availability and accessibility of the patient’s own GP for certain patient groups remains important (that is, for frail older people). Efforts should be made to improve personal continuity, focusing on the team-based provision of personal continuity.

## Method

### Study design and setting

We conducted a qualitative study using focus groups. Focus groups are able to stimulate discussion and generate a broad range of ideas using group dynamics and work well to collectively guide intervention development.^42^ We organised four focus groups from May 2019 to August 2019: two with GPs, one with patients and one with practice assistants and practice nurses. We chose unmixed groups as hierarchical differences between participants may hinder open group discussion. The number of focus groups was limited to four owing to constraints relating to time, research staff, and financial means. The focus groups were all set in the west of the Netherlands. Table 1 shows more details on participant characteristics.

**Table 1.** Participant characteristics

**Characteristic**	**FG1, GPs (*n* = 6)**	**FG2, GPs (*n* = 11)**	**FG3, patients (*n* = 7)**	**FG4, practice assistants and practice nurses (*n* = 6)**
**Job role**				
GP partner	2	4		
Locum GP	2	1		
Salaried GP	2	6		
Practice assistant				4
Practice nurse				2

**Age, years**				
≤30	0	0	0	2
31–49	3	2	0	1
50–66	2	9	0	3
≤67	1	0	7	0

**Sex**				
Male	2	7	1	0
Female	4	4	6	6

**Work experience, years**				
<10	0	1		2
10–20	3	3		3
21–30	2	6		1
>30	1	1		0

**Direct patient care per week, FTE**				
≤0.3	0	1		0
0.4–0.7	3	5		4
0.8–1.0	3	5		2

**Practice style**				
Two handed	1	1		0
Group practice	5	10		6

**Period registered at practice, years**				
<5			1	
5–10			2	
>10			4	

**Living situation**				
Alone			5	
Widowed			1	
Married/living together			1	

**Chronic diseases (besides vertigo), *n***				
0			2	
1–2			4	
>3			1	

*FG = focus group. FTE = full-time equivalent.*

### Procedure and participants

Focus group one (FG1) and two (FG2) were organised in June 2019 as part of an annual meeting for GP practices involved in research of Amsterdam UMC. During this meeting, GPs could choose to participate in several sessions or workshops. FG1 and FG2 were included as one of these sessions without preselection of participants. A sample of 17 GPs (FG1: *n* = 6, FG2: *n* = 11) was involved.

For focus group three (FG3), we recruited patients aged ≥65 years who had ≥1 chronic disease. Patients were included from an existing database from another study on chronic vertigo complaints.^43^^,^^44^ This database contained 490 patients aged ≥50 years. We included this patient group as a convenience sample as the patient database was available for our study and patients had previously given consent to be approached for participation in other research. From this database, 50 patients were sent a postal invitation accompanied by information on our study. Nine patients agreed to participate. Finally, seven patients participated in FG3, as two patients declined because of illness on the day of FG3.

Recruitment of practice assistants and practice nurses for focus group four (FG4) took place by inviting practice assistants (*n* = 67) and practice nurses (*n* = 49) from 14 practices in the Amsterdam area. In addition, the National Association of Practice Assistants distributed the invitation among its members using a newsletter. In total, four practice assistants and two practice nurses responded and agreed to participate in the focus group.

### Data collection

The focus groups were prepared by the first author and the senior author. All focus groups proceeded using the same structure. First, participants were invited to talk about their role in general practice (FG1, 2, 4) or about their experiences with general practice as a patient (FG3) and their experiences with personal continuity. Next, the first author presented a summary of the findings of our previous survey study, emphasising the barriers experienced by GPs and patients and the suggestions provided to improve personal continuity (see Supplementary Information S1 [in Dutch]). Participants were asked to reflect on the survey findings, to discuss their own experiences, and to provide suggestions on how they would improve personal continuity. These suggestions were further discussed for their feasibility and acceptability.

The focus groups were all led by an experienced moderator (the third author, the sixth author, and one other person [see acknowledgements]). The first author (FG1, FG3, and FG4) or senior author (FG2) was present as observer/note taker. Each focus group took 90 min, was audio-recorded, and transcribed verbatim after each session by a research assistant.

In October 2020, a member check was done by sending participants of the focus groups a report on their session for comments. Two GPs, five patients, two practice assistants and two practice nurses responded. Participants agreed with the report and emphasised the importance of team continuity to optimise personal continuity.

### Data analysis

Data was analysed using reflexive thematic analysis.^45^^–^47 Reflexive thematic analysis is an accessible qualitative research technique used to identify and analyse patterns in the data. It involves six phases. Phase one to three were performed by the first and second author who familiarised themselves with the data and independently generated initial codes without a previously defined codebook or theoretical framework. In phase four, the first and second author discussed the results to exchange views on the data and used codes and they drafted an initial thematic map. This thematic map was discussed between the first, third, fourth, sixth, seventh, eighth, and senior author and adjusted where necessary in phase five and included in this study as part of phase six. Atlas TI v24.0 software was used to organise and support data analysis. Our study was reported in accordance with the Standards for Reporting Qualitative Research checklist.^48^

## Results

### Definition

When discussing continuity, GPs, practice assistants, and practice nurses viewed personal continuity as being provided by the entire general practice team and not just by the patient’s own GP:

*‘We’re talking about personal continuity here. You just talked about team-based care, and I kind of agree with that* […]*. I’m not a GP all by myself. I’m a GP together with my assistants, my practice nurse, that’s with whom I run a general practice, that’s with whom I have to provide care.’*

(GP 4, FG1, aged 50–66 years, >30 years experience as a GP)

In this setting, the role of the patient’s own GP has changed. Instead of being the general provider of personal continuity of care for all patients, GPs, practice assistants, and practice nurses viewed having one own GP only necessary for certain patient groups:

*‘I believe continuity is essential for a selection of your registered patients, especially for frail older persons, I feel, or maybe complex psychiatry, oncology, or chronically ill patients.’*

(GP 5, FG1, aged 31–49 years, 10–20 years experience as a GP)

Although patients acknowledged the need for practice assistants and practice nurses, their own GP remained a central figure in their concept of personal continuity:

*‘If you have a regular GP, then … then you feel more comfortable. And that in turn has to do with knowing one another a little and … wanting to have trust in my GP.’*

(Patient 7, FG3, aged 70–74 years, registered with practice for >10 years, 1–2 chronic diseases)

### Challenges and potential solutions

All participants perceived that it has become increasingly difficult to provide or receive personal continuity in general practice. Contemporary general practices were viewed as larger-scale organisations with more patients and more GPs, who adopt part-time schedules, and were viewed to have a higher workload now compared with the past. Within this new setting, all participants acknowledged the importance of team communication and team stability to ensure that relevant patient information reaches the right team members and that the patient can trust each team member to have knowledge of the relevant medical information. In addition, all participants felt it was needed to stimulate the availability and accessibility of the patient’s own GP. An overview of the suggestions and barriers are summarised in Box 2. These viewpoints are elaborated on in the following paragraphs.

**Box 2.** Summary of thematic analysis results: participants’ suggestions and perceived barriers and facilitators for improving team communication and team stability and availability and accessibility of own GP

**Themes**	**Barriers (–) and/or facilitators (+)**
**Team communication and stability**	
*Team communication*	
Improve usage of EHR^a,b,c^ Have regular briefings on patients of interest^c^	Patients feel knowledge of EHR is (+)/is not (–) equivalent to having personal contact^b^
	There is sufficient (+)/insufficient (–) time available to do briefings^c^
*Team stability*	
Reduce practice size^a,b^ Reduce staff turnover by improving working conditions^a,c^ Reduce staff turnover by contractual obligation^a^	Payment system facilitates (+)/does not facilitate (–) practice size reduction^a^
	Suggestion was not further discussed
	There is no (+)/there is (–) a general staff shortage^a,c^

**Availability and accessibility of the patient’s own GP**	
*GP part-time work*	
• Make duos of part-time working GPs^a,c^	Suggestion was not further discussed
*GP burden*	
• Delegate tasks^a,b,c^	Patients know (+)/do not know (–) what the function is of practice healthcare workers^b,c^
	GPs can (+)/cannot (–) let go of their involvement in organisation^b,c^
	GPs perceive further task division as beneficial (+)/not beneficial (–)^a^
	There is staff available (+)/unavailable (–) to delegate to^a,c^
	There are sufficient (+)/insufficient (–) financial resources to hire a practice manager^a^
*Scheduling appointments*	
• Improve triage by practice assistants^a,b^	Practice assistant education level is sufficient (+)/insufficient (–) for triage^a^
	There is sufficient (+)/insufficient (–) time to perform triage^c^
• Give patients own responsibility in organising their care^a,c^	Patients have sufficient (+)/insufficient (–) digital skills^a,b^
	GPs can (+)/cannot (–) let go of their involvement in patient care^b,c^

a *GP’s perspective.*

b *Patient’s perspective.*

c *Practice assistant and practice nurses’ perspective. GP = general practitioner, EHR = electronic health record.*

### Team communication and team stability

Challenges for team communication and stability were mainly perceived by GPs and practice nurses and on the scale of practice organisation and staff turnover.

GPs, practice assistants, and practice nurses experienced a challenge in keeping a small, dedicated team within the scale of a larger organisation. They felt having a small team would be beneficial to personal continuity:

*‘It’s just short lines of communication. You can consult with each other very easily, you just know how the other works and that also makes it easy to communicate.’*

(GP 7, FG1, aged 50–66 years, 10–20 years experience as a GP)

Potential solutions were mainly seen in reducing practice size, improved usage of the electronic health record (EHR) and reducing staff turnover:

#### Improve usage of the EHR

Optimal use of the EHR was seen as indispensable for sharing data and keeping up to date by all participants. Patients also acknowledge the value of their EHR, yet they felt that it was of less value than having personal contact:

*‘The assistant … yes, they know my file, but they only look at that file but don’t know the person. If you only have one GP, they know exactly who you are. I miss that very much indeed.’*

(Patient 6, FG3, aged 70–74 years, >10 years registered with the practice, >3 chronic diseases)

#### Reduce staff turnover by facilitating a working environment

All participants perceived that a stable team enables healthcare workers to get to know their patients over time, improving personal continuity. GPs, practice assistants, and practice nurses suggested that staff turnover could be reduced by pleasant working conditions:

*‘I believe that if you organise your practice properly together, according to the wishes of the GP who performs the work by being flexible and facilitating combining work and private life,* […]*, it will be more pleasant for the GPs to work in your practice.’*

(GP 2, FG2, aged 50–66 years, 20–30 years experience working as a GP)

#### Reduce staff turnover by contractual obligation

One GP opted for a contractual obligation for new employees to work at least 5 years in the same practice to decrease locum work. However, other GPs and practice assistants mentioned that because of staff shortages there is no other option than to accept any available healthcare worker who was willing to work in their practice.

### Availability and accessibility of the patient’s own GP for patient care

Part-time working of GPs, increased GP burden, and the scheduling of appointments by practice assistants were seen as challenges by all participants for improving the availability and accessibility of the patient’s own GP for patient care.

Although all participants felt that decreasing part-time working would improve personal continuity, they also viewed it as infeasible in the context of contemporary general practice:

*‘*[…] *twenty years ago, you had solo GPs who worked 5 days a week. And they have disappeared. Apparently, there is something else for us as a professional group that means, that we don’t do that anymore and compromise on continuity of care because the work nowadays no longer aligns with working those 5 days.’*

(GP 10, FG2, aged 31–49 years, <5 years experience working as a GP)

GPs felt that the burden of the GP profession has increased because of more patient-related and administrative tasks. A patient compared this with similar problems in education and teaching:

*‘You actually have the same problem as in the past at primary schools … the head of a school who had always taught was no longer allowed to teach; they had to arrange everything. And so they became managers instead of teachers.’*

(Patient 4, FG3, aged 75–79 years, registered with the practice 5–10 years, 1–2 chronic diseases)

In general practice, practice assistants schedule appointments for patients, preferably with their own GP. However, appointment schedules are constrained because of increased GP burden and part-time working, while practice assistants also experience a high workload. Participants all experienced that this leads to practice assistants putting less priority on scheduling appointments with the patient’s own GP.

#### Make duos of part-time working GPs

Participants felt it was not feasible to increase GP working hours. Instead, participants suggested using ‘duo-doctors’: two part-time GPs sharing the same patient population and enabling a 5-day availability together:

*‘Our patients are registered with one GP. When patients come for introduction, I tell them they can visit my duo partner when I’m not there* [at the practice ...] *So you basically have your own GP, we encourage asking as much as possible for that person, and if the need is urgent, my colleague is also there.’*

(GP 6, FG2, aged 50–66 years, 20–30 years experience working as a GP)

### Delegate tasks

To decrease GP workload, all participants felt that GPs could delegate more tasks and should prioritise patient-related tasks. Administrative tasks could be performed by a practice manager and certain patient-related tasks could be done by other team members such as the practice nurse.

GPs, practice assistants, and practice nurses mentioned patients do not always know the role of general practice workers and are reluctant to accept the role of other team members. This was illustrated in the patient focus group:

*‘Yes, I find “practice nurse” incredibly confusing. Because I was referred to a practice nurse, who turned out to be a psychologist; well I had absolutely no need for that.* […] *So what are these practice nurses anyway? I thought they were just there to take blood samples.’*

(Patient 5, FG3, aged 70–74 years, registered with practice for 5–10 years, 1–2 chronic diseases)

Practice assistants and practice nurses noted that GPs can be reluctant to delegate tasks that could also be performed by them or a practice manager. A practice assistant reported a typical case:

*‘They* [GPs] *were working on* [installing] *a new coffee maker at our practice a few weeks ago, and then I think, like, you could have seen 20 patients!’*

(Practice assistant 5, FG4 aged 31–49 years, 10–20 years experience as a practice assistant)

GPs felt that delegating tasks could lead to loss of overview as their direct involvement in patient care decreases. This was illustrated by a GP discussing care for patients with chronic diseases, which is delegated to the practice nurse:

*‘Well, I believe the practice nurses also make things somewhat more difficult.* […] *I mean, you miss getting to know someone* [better] *which allows you to notice changes.’*

(GP 11, FG2, aged ≥67 years, 20–30 years experience working as a GP)

However, delegating care for frail older adults to a practice nurse specialised in this patient group was perceived as beneficial by GPs and patients:

*‘In our practice we have two to three hundred vulnerable older patients. That’s where the practice nurse for older patients is particularly useful. There is good communication, and they notice far more things.’*

(GP 2, FG2, aged 50–66 years, 20–30 years experience working as a GP)

#### Improve triage by practice assistants

GPs, practice assistants, and practice nurses viewed performing triage as essential for efficient planning. Practice assistants can use triage to assess urgency, consultation time needed, and to determine the need for a patient to see their own GP. As acute health issues or minor ailments do not require the patient’s own GP this could result in more efficient appointment time allocation.

GPs felt that triage has become too complex for practice assistants without any additional training*.* However, other GPs felt that, even with advanced training, triage by practice assistants could still be problematic:

*‘But the problem is that, for we’ve* [the GPs of the practice] *already been trying for years—I still believe it doesn’t work, it’s one of the most difficult parts of our profession which we actually leave to the assistants.’*

(GP 3, FG2, aged 50–66 years, 20–30 years experience working as a GP)

In addition, GPs and practice assistants, and practice nurses perceive a high workload and felt a lack of time for triage:

*‘Well, but I heard the other day of a practice where the workload is so high and with all those on sick leave and they just don’t do triage anymore, because there’s no time for it. They just have to make back-to-back appointments for 3 to 4 hours.’*

(Practice assistant 4, FG4, aged ≤30 years, <5 years experience as a practice assistant)

#### Give patients own responsibility in organising their care

To reduce practice assistants’ workload, the GPs, practice assistants, and practice nurses suggested enabling patients to schedule appointments online or to access their own EHR using digital tools. This would also allow patients to take responsibility over their own care. However, patients in our focus group perceived digital tools as unsuitable for an older population as they felt it was less personal. GPs felt that the lack of digital skills of patients could be a barrier for using digital tools. In addition, some GPs did not feel comfortable letting patients schedule their appointments themselves:

*‘If you have a somewhat larger centre and someone can choose from six GPs … those GPs don’t always find that desirable. So, you may well say, “the patient doesn’t mind seeing someone else,” but when the doctor says, “no, I actually want to provide continuity, so I don’t want my patients to be able to choose from six doctors”.’*

(GP 3, FG1, aged 50–66 years, 20–30 years experience as a GP)

## Discussion

### Summary

This qualitative study explored the perspectives of GPs, patients, practice assistants, and practice nurses on improving personal continuity and which barriers and facilitators affect the improvement process. Participants experienced that in current general practice, personal continuity of care is no longer exclusively provided by the patient’s own GP but by the entire general practice team. Patients having their own GP is still viewed as important, especially by older patients, but only necessary for certain patient groups (that is, those with chronic diseases or mental disorders). Participants suggested improving team communication (that is, by efficient use of EHR) and team stability (that is, by reducing staff turnover). It was suggested that to improve the accessibility and availability of a patient’s own GP for relevant patients, GP burden should be reduced by delegating tasks, working with duo-doctors, and appointments should be scheduled efficiently (that is, by optimal use of triage). Barriers and facilitators were perceived on the individual (for example, GPs involvement in organisation), organisational (for example, presence of staff shortages), or societal level (for example, payment system).

### Strength and limitations

A main strength of our study was the inclusion of different healthcare workers in general practice as well as patients. This allowed for a comparison of different perspectives on personal continuity. When combined with the results of our previous survey, we were able to improve the robustness of the findings.

Our focus group study took place in the Netherlands. The Netherlands is known to have a high-quality primary care system with a long tradition of personal continuity.^49^ The results of our focus group may therefore be used to align elements from other healthcare systems to the Dutch system to promote personal continuity.

Our method of sampling may influence the transferability of the results. Most GPs, practice assistants, and practice nurses were working in urban areas and all patients lived in an urban area. It is possible that some experiences are less transferable to more rural areas. The patients were included from a specific database on chronic dizziness. However, five out of seven patients had an additional chronic disease alongside vertigo, which could reduce potential bias.

We organised two focus groups for GPs, one focus group for patients, and one for practice assistants and practice nurses. A more comprehensive stakeholder involvement might have been obtained by including more than one focus group for each stakeholder group.

### Comparison with existing literature

Previously published interventions for improving personal continuity were either specific for certain payment systems, not systematically evaluated, or encountered implementation problems.^50^^–^53 Our study is, to our knowledge, the first to perform a qualitative assessment among stakeholders to collect suggestions for improving personal continuity in addition to the broad inventory of suggestions from our survey study.

The essence of personal continuity is often described as having a single regular GP who has accumulated knowledge over time about a patient.^26^^,^^54^ The different dimensions of continuity (personal, team, and cross-boundary continuity) do not stand alone and instead overlap.^3^^,^^55^ The GP, practice assistants, and practice nurses in our study in particular perceived that the distinction between personal continuity and team continuity has become less clear as the organisational structure of general practice continues to evolve. Therefore, personal continuity in general practice, as perceived by the study participants, could be best understood as a team of care providers who exchange relevant patient information, accumulate knowledge on patients together, and cooperate to ensure that the patient feels known and that care is coordinated.

Findings from earlier research on personal continuity align with the results of the present study. A mixed-methods study in Dutch general practice among GPs found that having a small and stable team, a smaller number of patients registered with one GP, and sufficient availability of the own GP for patient care may improve personal continuity.^56^ A qualitative study in Spain by Waibel *et al* (2018) found that adequate usage of the EHR, effective GP–patient communication, having a small-scale practice, and proactive GP involvement (for example, calling when a patient is admitted to a hospital) also have a positive influence on relational continuity.^57^ In this study, relational continuity was defined as an ‘ongoing therapeutic relationship with one or more providers spanning different healthcare episodes’.

Duo-doctors were seen as a possible solution for part-time working in our study. As fragmentation of the workforce because of part-time working and locum use was found to explain one-third of the decline of personal continuity, duo-doctors may be a valid strategy to improve personal continuity.^58^ A previous survey found that Dutch patients have no problem with having two own GPs, supporting the concept of duo-doctors in the Netherlands.^35^ Although no studies, to our knowledge, have evaluated the effectiveness of implementing duo-doctors on (personal) continuity, we know from studies in nursing settings that a similar concept can lead to, among others, less overtime, more work satisfaction, and more patient satisfaction.^59^ It is possible that a comparable effect may be observed after implementing duo-doctors in a general practice setting.

### Implications for research and practice

Although efforts for improving personal continuity in general practice should acknowledge the importance of patients having their own GP, emphasis should be put on strengthening the delivery of personal continuity by team-based care. As the provision of personal continuity of care is becoming more reliant on the general practice team, new opportunities arise to improve team-based provision of personal continuity. Some suggestions were viewed as acceptable with little or no barriers, such as working with duo-doctors, improved usage of the EHR, or giving patients more responsibility for organising their health care. As such, these suggestions may be prioritised for development into an intervention to improve personal continuity for older patients.

When improving health care, emphasis is often put on increasing resources and reducing staff shortages. Our study demonstrates that there are also barriers involving (lack of) knowledge (that is, patients’ knowledge about the function of practice healthcare workers) and the attitudes of both healthcare workers (that is, reluctance of GPs to delegate tasks) and patients (that is, aversion to triage) that could be addressed. There also seems to be a discrepancy between how health care is delivered in primary care according to the practice staff (that is, team-based care), and what patients expect to receive (that is, having one regular GP) that should be addressed. This discrepancy of views between practice staff and patients has not been adequately addressed in the literature so far and could be an interesting topic for further research.

In conclusion, this study provides new insights about how personal continuity in general practice might be improved. As general practice moves towards a team-based approach, efforts to improve personal continuity for older patients should focus on strengthening the team-based provision of personal continuous care.
